# Canonical Wnt signaling activation by chimeric antigen receptors for efficient cardiac differentiation from mouse embryonic stem cells

**DOI:** 10.1186/s41232-023-00258-6

**Published:** 2023-02-10

**Authors:** Takahiro Sogo, Shu Nakao, Tasuku Tsukamoto, Tomoe Ueyama, Yukihiro Harada, Dai Ihara, Tomoaki Ishida, Masato Nakahara, Koji Hasegawa, Yuka Akagi, Yasuyuki S. Kida, Osamu Nakagawa, Teruyuki Nagamune, Masahiro Kawahara, Teruhisa Kawamura

**Affiliations:** 1 Ritsumeikan Global Innovation Research Organization, Ritsumeikan University, 1-1-1 Noji-higashi, Kusatsu, Shiga 525-8577 Japan; 2 Department of Biomedical Sciences, College of Life Sciences, Ritsumeikan University, 1-1-1 Noji-higashi, Kusatsu, Shiga 525-8577 Japan; 3 Division of Translational Research, Kyoto Medical Center, National Hospital Organization, 1-1 Mukaihata-cho, Fukakusa, Fushimi-ku, Kyoto, 612-8555 Japan; 4 Department of Molecular Physiology, National Cerebral and Cardiovascular Center Research Institute, 6-1 Kishibe-Shimmachi, Suita, Osaka, 564-8565 Japan; 5 Cellular and Molecular Biotechnology Research Institute, National Institute of Advanced Industrial Science and Technology (AIST), Central 5-41, 1-1-1 Higashi, Tsukuba, Ibaraki 305-8565 Japan; 6 Tsukuba Life Science Innovation Program (T-LSI), School of Comprehensive Human Sciences, University of Tsukuba, 1-1-1 Tennoudai, Tsukuba, Ibaraki 305-8572 Japan; 7 School of Integrative & Global Majors, University of Tsukuba, 1-1-1 Tennoudai, Tsukuba, Ibaraki 305-8572 Japan; 8 Department of Bioengineering, Graduate School of Engineering, The University of Tokyo, 7-3-1 Hongo, Bunkyo-ku, Tokyo, 113-8656 Japan; 9 Laboratory of Cell Vaccine, Center for Vaccine and Adjuvant Research, National Institutes of Biomedical Innovation, Health, and Nutrition (NIBIOHN), 7-6-8 Saito-Asagi, Osaka Ibaraki City, 567-0085 Japan

**Keywords:** Canonical Wnt signaling, Cardiac differentiation, Chimeric antigen receptor, Pluripotent stem cells, Regenerative medicine

## Abstract

**Background:**

Canonical Wnt signaling is involved in a variety of biological processes including stem cell renewal and differentiation, embryonic development, and tissue regeneration. Previous studies reported the stage-specific roles of the Wnt signaling in heart development. Canonical Wnt signal activation by recombinant Wnt3a in the early phase of differentiation enhances the efficiency of myocardial cell production from pluripotent stem cells. However, the hydrophobicity of Wnt proteins results in high cost to produce the recombinant proteins and presents an obstacle to their preparation and application for therapeutics, cell therapy, or molecular analysis of Wnt signaling.

**Methods:**

To solve this problem, we generated an inexpensive molecule-responsive differentiation-inducing chimeric antigen receptor (designated as diCAR) that can activate Wnt3a signaling. The extracellular domains of low-density-lipoprotein receptor-related protein 6 (LRP6) and frizzeled-8 (FZD8) were replaced with single-chain Fv of anti-fluorescein (FL) antibody, which can respond to FL-conjugated bovine serum albumin (BSA-FL) as a cognate ligand. We then analyzed the effect of this diCAR on Wnt signal activation and cardiomyocyte differentiation of mouse embryonic stem cells in response to BSA-FL treatment.

**Results:**

Embryonic stem cell lines stably expressing this paired diCAR, named Wnt3a-diCAR, showed TCF/β-catenin-dependent transactivation by BSA-FL in a dose-dependent manner. Treatment with either Wnt3a recombinant protein or BSA-FL in the early phase of differentiation revealed similar changes of global gene expressions and resulted in efficient myocardial cell differentiation. Furthermore, BSA-FL-mediated signal activation was not affected by a Wnt3a antagonist, Dkk1, suggesting that the signal transduction via Wnt3a-diCAR is independent of endogenous LRP6 or FZD8.

**Conclusion:**

We anticipate that Wnt3a-diCAR enables target-specific signal activation, and could be an economical and powerful tool for stem cell-based regeneration therapy.

**Supplementary Information:**

The online version contains supplementary material available at 10.1186/s41232-023-00258-6.

## Background

Canonical Wnt signaling controls the stem cell properties of self-renewal and differentiation and is involved in embryonic development and tissue regeneration. During early vertebrate development, canonical Wnt signaling is required for mesoderm formation and it participates in anterior-posterior patterning, specifying the posterior mesodermal fates [1, 2]. Furthermore, previous studies reported the stage-specific roles of the Wnt signaling in cardiogenesis from embryonic stem (ES) cells. In the early phase of differentiation, the activation of canonical Wnt signaling by Wnt3a recombinant protein enhances the efficiency of myocardial cell production from mouse or human pluripotent stem cells [1–5].

Wnt3a-induced signaling cascade is initiated by binding of Wnt3a to the single-transmembrane (TM) protein low-density-lipoprotein receptor-related protein 6 (LRP6) and the seven-transmembrane protein frizzled 8 (FZD8) [6–8]. The formation of the Wnt3a/LRP6/FZD8 complex results in the recruitment of APC/Axin destruction complex at the cell membrane, thereby perturbing the phosphorylation and subsequent ubiquitination of β-catenin [9]. The stabilized β-catenin translocates into the nucleus where it interacts with T cell factor (TCF) and transactivates the downstream target genes [6–8, 10, 11]. However, Wnt proteins are considered problematic for their application to cell therapy and/or regenerative medicine. Production of high-quality recombinant Wnt proteins in a large scale is complicated due to their palmitoylation which makes them highly hydrophobic. Thus, recombinant Wnt proteins are too expensive to apply for mass production of cardiomyocytes differentiated from pluripotent stem cells at the industrial level [9]. To address this issue, we attempted to generate artificial receptors that can activate Wnt3a signaling in response to an inexpensive surrogate ligand.

We previously developed proliferation-inducing and migration-inducing chimeric antigen receptors (piCARs and miCARs), which we also called growth signalobodies and migration signalobodies, respectively [12–22]. In most of the piCARs and miCARs, an anti-fluorescein (FL) single-chain Fv (scFv) fused to the extracellular D2 domain of EpoR was joined to the TM and cytoplasmic domains of single membrane-spanning type I cytokine receptors or receptor tyrosine kinases. When FL-conjugated bovine serum albumin (BSA-FL) is used as a ligand, it triggers receptor oligomerization and activates signal transduction dependent on the cytoplasmic domain. Using the same concept, apoptosis-inducing chimeric antigen receptors (aiCARs), which we had called death signalobodies, were also developed using the signaling molecules involving in death-inducing signaling complex [23–25]. More recently, we have modified single membrane-spanning cytokine receptors and we have succeeded in creating differentiation-inducing chimeric antigen receptors (diCARs), which we called differentiation signalobodies [26, 27]. As cell differentiation is very important for creating various cell types in vivo, further development of more diverse series of diCARs is of utmost importance for producing specific cells desired for regenerative medicine. Although cell differentiation is regulated by a variety of receptor superfamilies including multiple membrane-spanning G protein-coupled receptors (GPCRs), all of the cell fate-inducing CARs developed to date were based on single membrane-spanning receptors.

In the present study, we, for the first time, developed GPCR-based CARs, which are able to activate endogenous Wnt3a signaling in mouse ES cells. We constructed a pair of chimeras, in which scFv of anti-FL antibody was tethered to the TM/cytoplasmic domains of LRP6 or FZD8. These chimeras are able to bind to BSA-FL and are expected to form BSA-FL/chimeric LRP6/chimeric FZD8 complexes mimicking the Wnt3a/LRP6/FZD8 complexes. We named this chimeric pair Wnt3a-diCAR and investigated its capability to transduce Wnt3a signaling in response to BSA-FL stimulation. Because BSA-FL is a stable molecule with no toxicity and it costs much less than recombinant Wnt3a, regulation of Wnt3a signaling by the Wnt3a-diCAR may contribute to highly efficient and economical myocardial creation from pluripotent stem cells.

## Methods

### Plasmid constructions

To construct the plasmids for the lentivirus vector preparation, pLenti-CAG-EGFP [28, 29] was modified. In brief, an EGFP sequence was replaced with an IRES sequence containing puromycin- or blasticidin-resistant gene, to create pLenti-CAG-IRES-Puro or pLenti-CAG-IRES-Blast, respectively. A cDNA fragment encoding anti-fluorescein scFv was amplified by PCR from pMK-S β-I-Sγ-IG [21] using PrimeSTAR HS DNA polymerase (TAKARA Bio). This fragment was digested with *Bst*BI and *Eco*RV and subcloned into pLenti-CAG-IRES-Puro and pLenti-CAG-IRES-Blast, resulting in pLenti-CAG-scFv-IRES-Puro and pLenti-CAG-scFv-IRES-Blast, respectively. The cDNAs encoding mouse LRP6 and FZD8 were obtained from total RNA of mouse heart by RT-PCR using SuperScript III Reverse Transcriptase (Life Technologies) and PrimeSTAR HS DNA polymerase (TAKARA Bio). The primers used for plasmid construction and RT-PCR are listed on Supplementary Table 1. Each amplified fragment of LRP6L or LRP6M was directly inserted into *Eco*RV-digested pLenti-CAG-scFv-IRES-Puro, and that of FZD8L was inserted into *Eco*RV-digested pLenti-CAG-scFv-IRES-Blast to create pLenti-CAG-scLRP6L-IRES-Puro, pLenti-CAG-scLRP6M-IRES-Puro, and pLenti-CAG-scFZD8L-IRES-Blast, respectively. To construct pLenti-CAG-scLRP6S- IRES-Puro and pLenti-CAG-scFZD8S-IRES-Blast, fragments of LRP6S and FZD8S were digested with *Age*I, and subcloned into pLenti-CAG-scFv-IRES-Puro and pLenti-CAG-scFv-IRES-Blast digested with *Bsp*EI, respectively.

### Cell culture, creation of stable cell lines, and in vitro differentiation

A mouse ES cell line, CGR8, derived from 129/Ola was maintained on gelatin-coated dishes in DMEM (Nacalai Tesque)-based ES cell culture medium containing 10% fetal bovine serum, 1 mM sodium pyruvate, MEM non-essential amino acids, 0.1 mM 2-mercaptoethanol, GlutaMAX (all from Life Technologies), nucleosides (Chemicon), penicillin/streptomycin (FUJIFILM Wako Pure Chemical), and 1000 U/ml LIF (Chemicon). For generating stable cell lines, ES cells were transduced with lentivirus vectors encoding the transgene and antibiotic-resistant gene. The cells were subsequently selected by antibiotics to create the antibiotic-resistant stable ES cell line co-expressing scLRP6S and either scFZD8S (ES/SLR6S/scFZD8S cell line) or scFZD8L (ES/SLR6S/scFZD8L cell line). Mouse ES cells were also transduced with a pair of mock vectors, pLenti-CAG-IRES-Puro and pLenti-CAG-IRES-Blast, as a negative control cell line (ES/mock). ES cell differentiation experiments were performed through embryoid body (EB) formation, as previously described [26, 30]. In brief, cells were dissociated using 0.05% trypsin/EDTA (FUJIFILM Wako Pure Chemical) and subjected to a hanging drop method (750 cells/drop in the ES cell culture medium without LIF). Six days after EB formation, EBs were transferred into a gelatin-coated 48-well plate (one EB/well) and cultured in an attachment condition. We monitored the attached EBs under a microscope and counted the numbers of spontaneously beating clusters. EBs were stimulated with Wnt3a, Dkk1 (both from R&D systems), fluorescein, BSA-FL (both from Sigma), or vehicle for the indicated time. For the culture of NIH3T3 cells or mouse embryonic fibroblasts (MEF), these cells are maintained in DMEM containing 10% fetal bovine serum, GlutaMAX (Life Technologies), and penicillin/streptomycin (FUJIFILM Wako Pure Chemical).

### Transient transfection and luciferase assays

Cells were cultured on a gelatinized 48-well plate (6 × 10^4^ cells/well) for 24 h and subsequently transfected with a TOPflash firefly luciferase reporter plasmid [31] and a pSV40-Rluc control plasmid using Fugene HD (Roche) in an antibiotic-free medium supplemented with 1000 U/ml LIF. After 24 h, the medium was changed with fresh one containing Wnt3a, BSA-FL, fluorescein, CHIR99021 (FUJIFILM Wako Pure Chemical), Dkk1, XAV939 (Selleckchem), IWP-4 (Cayman Chemical), or vehicle. Following 24-h culture, cells were lysed and used for the luciferase assay, using PicaGene Dual Sea Pansy Luminescence Kit (FUJIFILM Wako Pure Chemical). Luminescence intensity was measured by ARVO Multilabel Plate Reader (PerkinElmer). The firefly luciferase activity was normalized to the Renilla luciferase activity, used as a marker of transfection efficiency.

### Western blotting

Cultured cells were lysed in 2× SDS-containing buffer, and the protein concentrations were quantified using BCA assay kit (Takara Bio). Protein samples were then reduced and boiled. Subsequently, equal amounts of protein were applied into each lane and separated in an 8% polyacrylamide tris glycine SDS gel, then transferred to a nitrocellulose membrane. The membranes were blocked in 2% skim milk-containing TBS supplemented with 0.1% Tween-20 for 1 h at room temperature and incubated with primary antibodies overnight at 4 °C. Following a washing step, the membranes were incubated with HRP-conjugated secondary antibodies. Signals were developed using Pierce ECL Western Blotting Substrate (Thermo Fisher Scientific), and acquired with Image Quant LAS 4000 mini (GE Healthcare). Antibodies used in this study were rabbit polyclonal anti-Ror2 antibodies (BD Biosciences), mouse monoclonal anti-β-actin antibodies (Sigma-Aldrich), anti-rabbit IgG-HRP goat antibodies (Jackson), and anti-mouse IgG-HRP antibodies (Jackson).

### Immunofluorescence

Based on the standard protocol of immunofluorescence labeling as previously described [26, 30], EBs differentiated from ES/scLRP6S/scFZD8L were fixed and stained with anti-cardiac α-actinin primary antibody (Abcam). Signals were visualized by anti-mouse IgG-Alexa594 secondary antibody (Life Technologies), and nuclear counterstain was performed using 4’,6-diamidino-2-phenylindole (DAPI, Life Technologies). Images were acquired using a BZ-X710 fluorescence microscope and a BZ-X software (Keyence). For BSA-FL detection, EBs differentiated from ES/mock and ES/scLRP6S/scFZD8L on day 3 with vehicle or BSA-FL treatment were fixed and incubated in 10%/20%/30% sucrose/PBS series. Frozen sections were collected from these EBs embedded in OCT compound (Sakura Finetek Japan) and counterstained in DAPI-containing PBS. Confocal images were obtained with TCS SP5 confocal microscope and LAS-AF software (Leica Microsystems).

### DNA microarray

Total RNA was isolated using a TRIzol reagent (Life Technologies), and sense-strand cDNA samples were prepared using Ambion WT Expression Kit (Life Technologies). The samples were hybridized in GeneChip Mouse Gene 1.0 ST Array (Affymetrix) following the manufacturer’s instructions. Hybridized arrays were imaged using GeneChip Scanner (Affymetrix), and the data were analyzed by an Expression Console software (Affymetrix) for differential expression among the examined conditions. The similarity of the gene expression profiles among the datasets was measured by Pearson’s correlation coefficient analysis. Hierarchical clustering was performed for heatmap visualization using Cluster 3.0 software. GO enrichment analysis was performed using the Database for Annotation, Visualization, and Integrated Discovery (DAVID) software.

### Quantitative RT-PCR

Total RNA was isolated using the TRIzol reagent, and cDNA was synthesized by High Capacity cDNA Reverse Transcription Kit. StepOnePlus Real-time PCR System (Life Technologies) and SYBR Premix Ex Taq II (Takara Bio) were used for quantitative PCR. Gene expression levels of target genes were normalized to the levels of *Gapdh*. Gene-specific primers used for quantitative PCR are listed on Supplementary Table 2.

### Real-time luciferase assays

ES/mock and ES/SLR6S/scFZD8L stably expressing the TOPflash reporter gene were created and stimulated with Wnt3a, BSA-FL, or vehicle from day 2 to day 3 of differentiation. The resulting EBs were transferred into low attachment dishes, and luciferase activities in 50 live EBs were measured by a Kronos Dio luminometer (ATTO) every 10 min from day 3 to 8 of differentiation. All measured values were corrected to estimated cell numbers at the same time points. The cell numbers from days 3 to 8 were calculated by Gompertz curve or Logistic curve using actual cell numbers of 15 to 20 EBs counted on days 3, 5, 6, and 8.

### Fluorescence-activated cell sorting

EBs from ES/mock or ES/scLRP6S/scFZD8L stimulated with vehicle or 1 μg/ml BSA-FL were dissociated with 0.25% trypsin/EDTA on day 10 of differentiation. Cells were fixed, permeabilized, and then immunolabeled with anti-cardiac troponin T (cTnT) mouse monoclonal antibodies (abcam). Anti-mouse IgG antibodies conjugated with Alexa Fluor 488 (Life Technologies) were used as secondary antibody. After cells were washed and filtrated through a cell strainer, cTnT^+^ cells were quantified with FACS Aria III cell sorter and FlowJo software (BD Biosciences).

### Calcium imaging

EBs of ES/scLRP6S/scFZD8L were stimulated with 1 μg/ml BSA-FL from day 2 to day 3 and transferred into gelatinized dish on day 6 of differentiation. On day 17, the cells were stained with Fluo-8 AM calcium indicator (AAT Bioquest) according to the manufacturer’s instructions. Time-lapse images of calcium oscillation were acquired using BZ-X710 fluorescence microscope and BZ-X software (Keyence). Signal intensities were quantified using ImageJ software.

### Statistics

For statistical analysis, Student’s *t* test was used to compare the mean between two groups. Tukey’s multiple comparison test was performed to analyze the differences among the results of three groups. All data are expressed as mean ± SD.

## Results

### Chimeric antigen receptor construction and its Wnt3a signal transduction

**Fig. 1:**
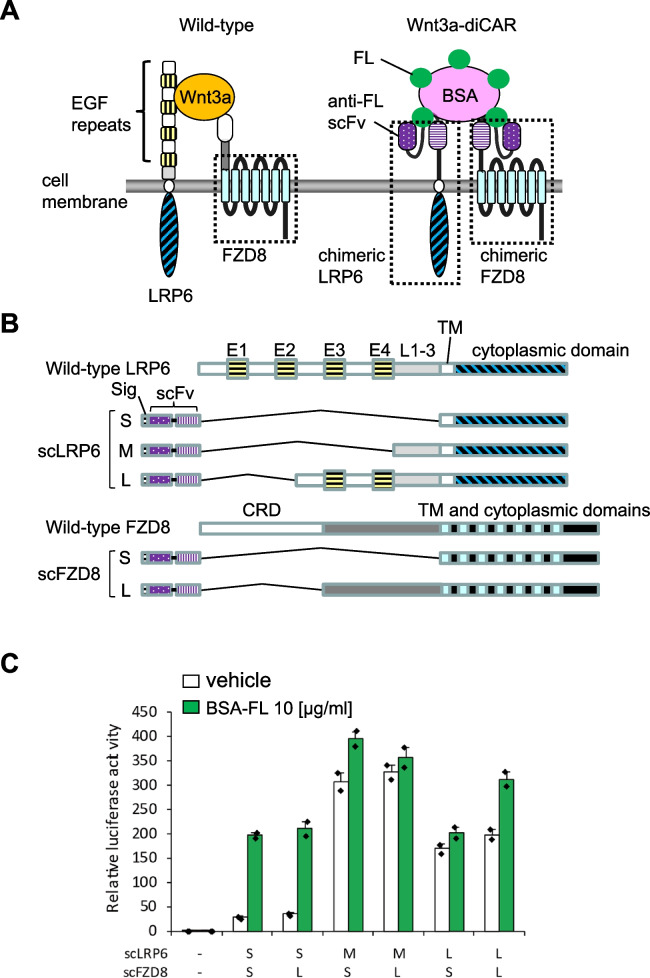
Construction of the Wnt3a-diCARs and their signal transduction property in mouse ES cells. **A** A schematic illustration of wild-type and the chimeric antigen receptors for Wnt3a (Wnt3a-diCAR). Wnt3a-diCAR consists of the single-chain Fv region (scFv) of an anti-fluorescein (FL) antibody and the transmembrane/cytoplasmic domains of LRP6 and Frizzled8 (FZD8). **B** Molecular structures of wild-type and chimeric LRP6 and FZD8 proteins. An immunoglobulin heavy chain secretion signal sequence (Sig) is placed upstream of each chimera for cell surface expression. The EGF repeats 1–4 (E1–E4), LDLR repeats 1–3 (L1–3), cysteine-rich domain (CRD), and transmembrane domain (TM) are indicated. **C** TCF/β-catenin-dependent transcriptional activities were measured by luciferase reporter assays in mouse ES cells transiently transfected with various combinations of chimeric LRP6 and FZD8. The signal was normalized to the vehicle-treated mock-transfected cells, which was set to 1. The data represent the mean of duplicates and standard deviation from one representative experiment

To activate Wnt3a signaling via diCARs, we designed and constructed antibody/LRP6 chimeras and antibody/FZD8 chimeras (Fig. 1A, B). These chimeras contain the scFv region of an anti-FL antibody, which allows the recognition of an FL molecule as a cognate ligand. We constructed three LRP6 chimeras (named scLRP6S, scLRP6M, and scLRP6L) and two FZD8 chimeras (named scFZD8S and scFZD8L, Fig. 1B). The extracellular domain of wild-type LRP6 consists of four EGF repeats and three LDLR repeats [32, 33]. The first two EGF repeats were required for the ligand binding to LRP6 [34]. Conformational differences among these candidate chimeras composing the Wnt3a-diCAR are as follows: the whole extracellular domain, all the EGF repeats, or the first two EGF repeats of wild-type LRP6 was deleted, and the anti-FL scFv region was placed to form scLRP6S, scLRP6M, or scLRP6L, respectively. The cysteine-rich domain (CRD) of wild-type FZD8 is also required for generating Wnt3a/LRP6/FZD8 complex [6]. The whole extracellular domain or the CRD of wild-type FZD8 was replaced with the anti-FL scFv region in scFZD8S or scFZD8L, respectively.

To examine their signal transduction properties, several combinations of scLRP6 and scFZD8 constructs were transiently transfected into mouse ES cells. Activities of TCF/β-catenin-dependent transcription, a convergent downstream target of Wnt3a signaling, were subsequently measured by a luciferase reporter assay (Fig. 1C). We used BSA-FL as a ligand, because multiple FL molecules of BSA-FL would facilitate receptor oligomerization and trigger Wnt3a signaling. TCF/β-catenin transactivation was observed in cells expressing any examined pairs of chimeras with or without BSA-FL. In particular, transfection of scLRP6M/scFZD8S, scLRP6M/scFZD8L, scLRP6L/scFZD8S, or scLRP6L/scFZD8L pairs showed ligand-independent transactivation. However, scLRP6S/scFZD8S- or scLRP6S/scFZD8L-expressing cells exhibited BSA-FL-mediated activation with low basal signal activity. These findings indicate that BSA-FL, a surrogate ligand, may control Wnt3a/β-catenin signal transduction in mouse ES cells expressing either scLRP6S/scFZD8S or scLRP6S/scFZD8L.

### Mouse ES cells stably expressing the Wnt3a-diCARs exhibit Wnt3a signal activation upon BSA-FL stimulation

**Fig. 2:**
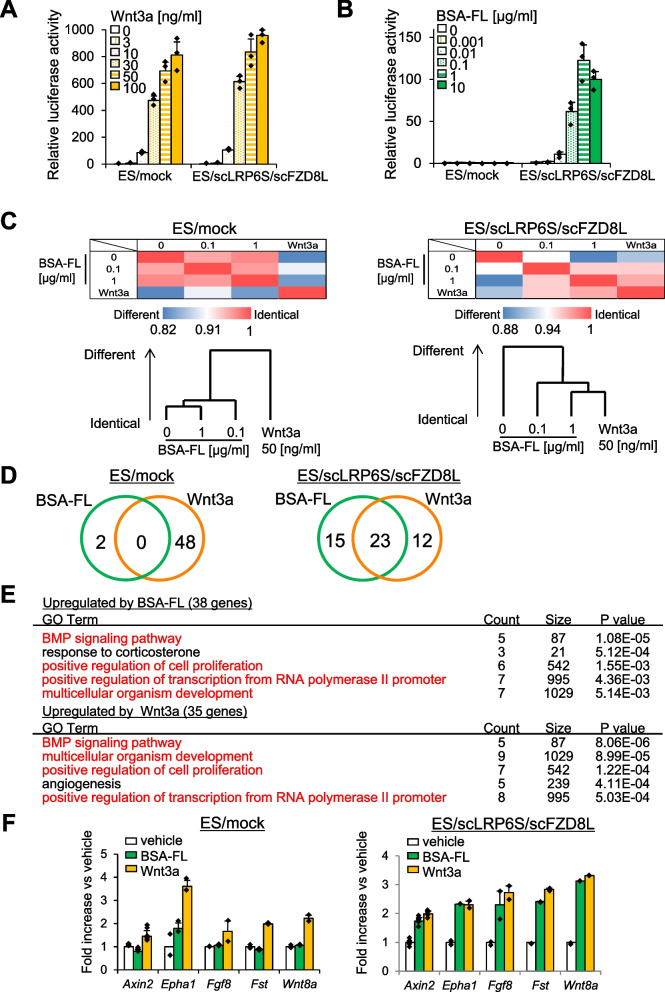
BSA-FL treatment activates TCF/β-catenin-dependent transcription and upregulates the expression of Wnt target genes in ES/scLRP6S/scFZD8L. **A, B** TCF/β-catenin-dependent luciferase activities in ES/mock and ES/scLRP6S/scFZD8L in response to various concentrations of BSA-FL (**A**) or Wnt3a (**B**). Firefly luciferase activities were normalized to Renilla luminescence intensities driven by the constitutively active promoter. The values in ES/mock treated with vehicle were set to 1. The data represent the mean of triplicates and standard deviation from one representative experiment. **C** A heatmap of Pearson’s correlation coefficient and dendrogram among DNA microarray data from ES/mock and ES/scLRP6S/scFZD8L stimulated with 0, 0.1, or 1 μg/ml BSA-FL, or 50 ng/ml Wnt3a from days 2 to 3 of differentiation. **D** Venn diagram showing the number of upregulated genes by 1 μg/ml BSA-FL (green) or 50 ng/ml Wnt3a (orange) in ES/mock and ES/scLRP6S/scFZD8L. **E** GO analysis of the 38 genes upregulated by 1 μg/ml BSA-FL and 35 genes upregulated by 50 ng/ml Wnt3a in ES/scLRP6S/scFZD8L are shown. Red characters indicate the overlapped categories. **F** Expression levels of Wnt target genes on day 3 of differentiation of ES/scLRP6S/scFZD8L. Cells were stimulated with vehicle, 0.1 μg/ml BSA-FL, or 50 ng/ml Wnt3a from day 2 to day 3. Expression levels of vehicle-treated cells were set to 1. The data represent the mean of duplicates and standard deviation from one representative experiment

To examine the functional role of the Wnt3a-diCAR in ES cells, we transduced them with a pair of lentiviral vectors. We established the stable ES cell line co-expressing scLRP6S and either scFZD8S or scFZD8L, named ES/scLRP6S/scFZD8S or ES/scLRP6S/scFZD8L, respectively. A pair of mock vectors, which do not carry the diCAR genes, was also used to generate the negative control cell line (ES/mock). Then, we performed the TCF/β-catenin-dependent luciferase reporter assay to determine whether these ES cell lines exhibit Wnt3a signal activation in response to BSA-FL stimulation. ES/scLRP6S/scFZD8S showed only a modest TCF/β-catenin transactivation in response to BSA-FL, and ES/mock displayed no response (Fig. S1). However, ES/scLRP6S/scFZD8L displayed dose-dependent transactivation by BSA-FL stimulation (Fig. 2B). In contrast to the results from the transient transfection experiment (Fig. 1C), ligand-independent signal activation was not observed in the corresponding stable cell line (Fig. 2B). Moreover, there was no significant difference between ES/mock and ES/scLRP6S/scFZD8L abilities to mediate Wnt3a signaling upon the administration of recombinant Wnt3a or CHIR99021, a canonical Wnt activator (Fig. 2A, Fig. S2). We also verified that BSA-FL increased TCF/β-catenin-dependent transactivation in Wnt3a-diCAR-expressing MEFs (Fig. S3). In addition, to examine the effect of this diCAR on non-canonical Wnt activation, we performed Western blotting to detect the protein expression of Ror2, a corresponding non-canonical Wnt receptor. In contrast to treatment with recombinant Wnt5a, a non-canonical Wnt ligand, BSA-FL stimulation did not affect Ror2 protein levels in mouse ES cells and fibroblasts. Of note, changes of Ror2 expression in response to BSA-FL were similar to those in a Wnt3a-treated condition (Fig. S4).

Using DNA microarray and quantitative RT-PCR analyses, we next examined BSA-FL-induced changes of the mRNA expression profile of ES/mock and ES/scLRP6S/scFZD8L. BSA-FL treatment and Wnt3a treatment in ES/scLRP6S/scFZD8L resulted in similar mRNA expression profile, while global mRNA expression levels of BSA-FL-treated ES/mock and vehicle-treated cells were similar (Fig. 2C). As for the top 50 genes upregulated by Wnt3a or BSA-FL administration, they were completely differential in ES/mock, whereas approximately half of the genes were overlapped in ES/scLRP6S/scFZD8L (Fig. 2D). This overlapping was exemplified by a gene ontology (GO) analysis based on genes upregulated by BSA-FL or Wnt3a treatment in ES/scLRP6S/scFZD8L. Both treatments increased the expression level of genes enriched for similar GO terms (indicated by red characters) associated with ES cell differentiation, including BMP signaling pathway, positive regulation of cell proliferation, and multicellular organism development (Fig. 2E). Furthermore, the target genes of Wnt3a signaling, Axin2, Epha1, Fgf8, Follistatin, and Wnt8a, were selected based on our DNA microarray analysis and previous reports [34, 35]. These genes were significantly upregulated in BSA-FL- and Wnt3a-treated ES/scLRP6S/scFZD8L, but not in BSA-FL-treated ES/mock (Fig. 2F). The above results suggest that ES/scLRP6S/scFZD8L has a potential to specifically activate Wnt3a signaling by administration of a surrogate ligand, BSA-FL.

### Myocardial differentiation from ES/scLRP6S/scFZD8L is promoted by BSA-FL

**Fig. 3:**
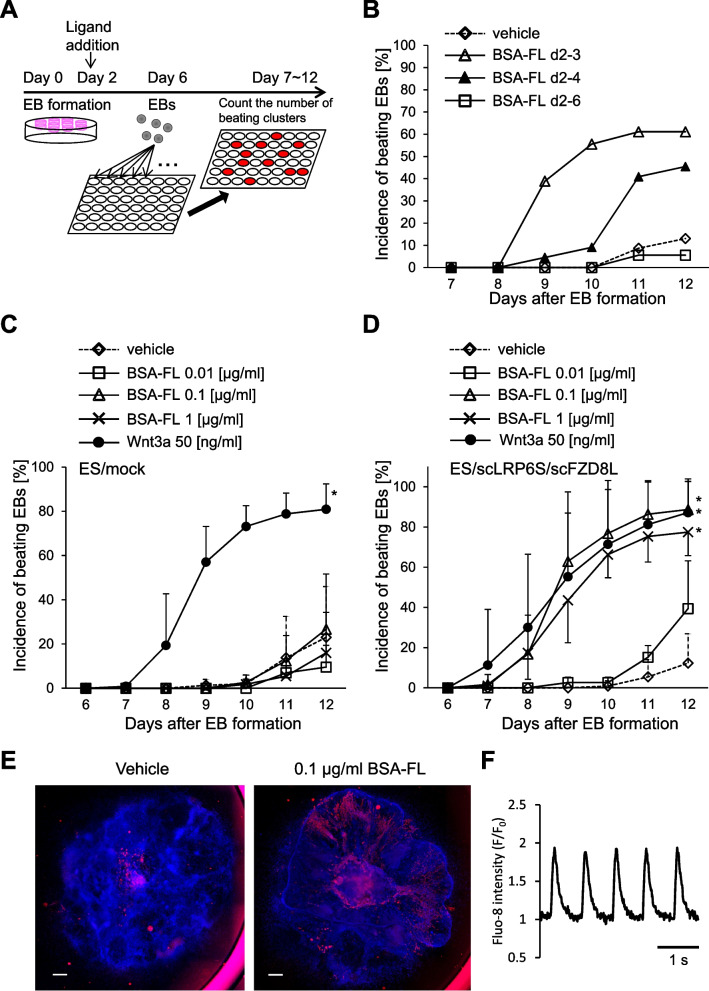
BSA-FL induces efficient myocardial differentiation from ES/scLRP6S/scFZD8L. **A** An illustration of a myocardial differentiation procedure for mouse ES cells used in this study. **B** The incidence of spontaneously beating EBs derived from ES/scLRP6S/scFZD8L from day 7 to day 12 of differentiation. Cells were stimulated with 0.1 μg/ml BSA-FL from day 2 to day 3, day 2 to day 4, or day 2 to day 6 as indicated. The data are shown from a representative experiment. **C, D** Time course of the incidence of spontaneously beating EBs from ES/mock (**C**) and ES/scLRP6S/scFZD8L (**D**) stimulated by various concentrations of BSA-FL, 50 ng/ml Wnt3a, or vehicle for 24 h from day 2 after EB formation. The data represent the mean and standard deviation from 3 to 10 independent experiments. *p < 0.05 (vs vehicle; significance was assessed by Turkey’s multiple comparison test). **E** Representative immunofluorescence images of EBs derived from ES/scLRP6S/scFZD8L treated with vehicle (left) or BSA-FL (middle) are shown. An anti-cardiac α-actinin antibody was used to detect cardiomyocytes (red) and DAPI was used for nucleus staining (blue, scale bars, 500 μm). **F** Representative trace of the intracellular Ca^2+^ change in the beating cluster as indicated by fluorescence intensity

Wnt/β-catenin signaling is involved in mesodermal formation and cardiogenesis. Thus, we examined the role of BSA-FL-dependent Wnt3a signal activation in in vitro differentiation of ES cells. ES/mock and ES/scLRP6S/scFZD8L were subjected to EB formation using a hanging drop method (Fig. 3A). EBs were then treated with BSA-FL. Confocal imaging revealed that BSA-FL diffused into EBs (Fig. S5). The efficiency of myocardial differentiation was assessed by quantifying the incidence of spontaneously beating EBs among all the examined EBs. To determine the time and duration of BSA-FL stimulation for myocardial differentiation, EBs derived from ES/scLRP6S/scFZD8L were stimulated with 0.1 μg/ml BSA-FL for 24, 48, or 96 h from day 2 of differentiation. The highest incidence of spontaneous beating EBs was scored in the EBs stimulated with BSA-FL for 24 h, whereas longer BSA-FL stimulation resulted in lower incidence (Fig. 3B). These findings are consistent with previous studies demonstrating that myocardial differentiation is promoted when ES cells are stimulated with Wnt3a only in the early stage of differentiation [1, 2]. Optimal BSA-FL dose was determined in EBs from ES/mock and ES/scLRP6S/scFZD8L stimulated between days 2 and 3 of differentiation with 0.01, 0.1, or 1 μg/ml BSA-FL. The BSA-FL concentrations, which most effectively induced spontaneously beating ES/scLRP6S/scFZD8L-derived EBs, were 0.1 and 1 μg/ml. The maximal induction rates after BSA-FL-treatment and Wnt3a-treatment were similar (Fig. 3C, D). In contrast, the incidence of beating EBs was not increased in ES/mock treated with BSA-FL (Fig. 3C). Similar results were obtained using EBs differentiated from three other independent ES cell lines expressing Wnt3a-diCAR (Fig. S6). Furthermore, immunofluorescence staining revealed that the positive area of a cardiac marker, cardiac α-actinin, was larger in EBs derived from ES/scLRP6S/scFZD8L stimulated with BSA-FL than in vehicle-treated EBs (Fig. 3E). To quantify the number of cardiomyocytes differentiated from ES/mock and ES/scLRP6S/scFZD8L cells with or without BSA-FL treatment, we performed flow cytometric analysis. Then, we found that BSA-FL increased a cardiomyocyte population in ES/scLRP6S/scFZD8L cells as observed in cells treated with recombinant Wnt3a (Fig. S7). We also confirmed that cardiomyocytes derived from BSA-FL-stimulated ES/scLRP6S/scFZD8L showed regular oscillations of intracellular calcium (Fig. 3F and Movie S1).

**Fig. 4:**
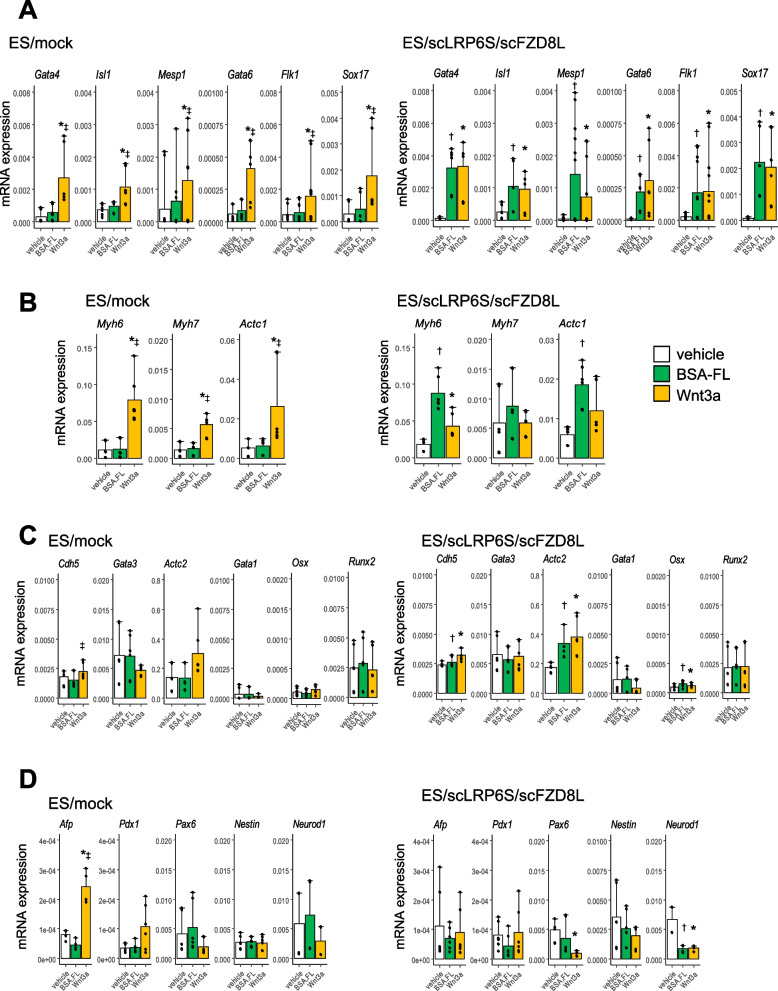
Quantitative gene expression analysis of mesodermal progenitor and differentiation markers in EBs derived from ES/scLRP6S/scFZD8L. **A–D** mRNA expression levels of mesodermal progenitor markers on day 6 (**A**), cardiac and non-cardiac mesodermal markers (**B, C**), and endodermal markers and ectodermal cell markers (**D**) on day 13. EBs were derived from ES/mock and ES/scLRP6S/scFZD8L stimulated with vehicle, 0.1 μg/ml BSA-FL, or 50 ng/ml Wnt3a from day 2 to day 3 of differentiation. The expression levels were normalized with those of an internal control (Gapdh). The data represent the mean and standard deviation from 3 to 6 independent experiments. *p < 0.05 (vehicle vs Wnt3a). †*p* < 0.05 (vehicle vs BSA-FL). ‡*p* < 0.05 (BSA-FL vs Wnt3a). Significance was assessed by Turkey’s multiple comparison test

To further examine the preferential cell differentiation accelerated by BSA-FL in ES/scLRP6S/scFZD8L, we evaluated the expression levels of mesodermal, ectodermal, and endodermal marker genes in the differentiated EBs in the mid-phase (day 6) and late-phase (day 13) of differentiation. The expression levels of mesodermal progenitor markers (Gata4, Isl1, Mesp1, Gata6, Flk1 and Sox17) (Fig. 4A), and cardiac markers [Myh6 (α-MHC), Myh7 (β-MHC) and Actc1 (α-cardiac actin)] (Fig. 4B), were all upregulated in BSA-FL-stimulated EBs from ES/scLRP6S/scFZD8L. In contrast, the changes of the expression levels of non-cardiac mesodermal marker genes [Cdh5 (VE-cadherin), Actc2 (α-smooth muscle actin), Gata1, Gata3, Osx, and Runx2], endodermal marker genes (Afp and Pdx1), and ectodermal marker genes (Pax6, Nestin, and Neurod1) were not significantly different between EBs from ES/mock and ES/scLRP6S/scFZD8L after treatment with BSA-FL or Wnt3a (Fig. 4C, D). These findings indicate that ES/scLRP6S/scFZD8L are efficiently differentiated into cardiomyocytes rather than other cell types in response to BSA-FL as an activator of Wnt3a signaling.

### Wnt3a-diCAR-dependent signaling is inhibited by monomeric fluorescein but not by Dkk1

**Fig. 5:**
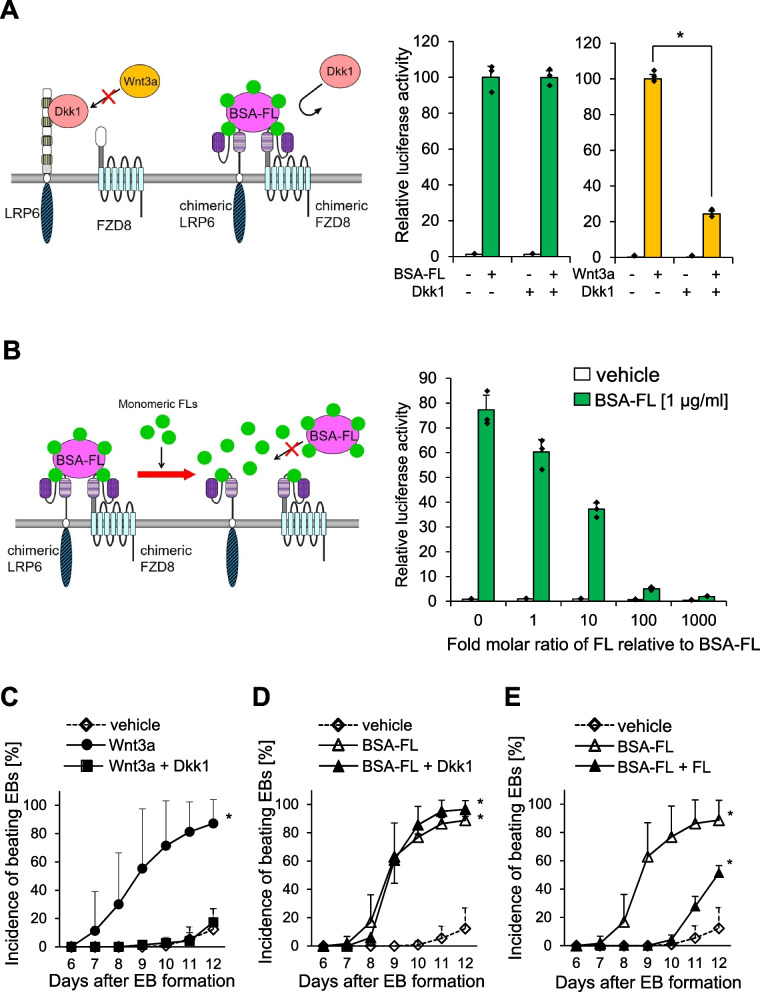
Antagonizing Wnt3a- and BSA-FL-dependent signaling suppressed TCF/β-catenin-dependent transcription and myocardial differentiation from ES/scLRP6S/scFZD8L. **A, B** TCF/β-catenin-dependent luciferase reporter activities in ES/scLRP6S/scFZD8L stimulated with vehicle, 0.1 μg/ml BSA-FL or 50 ng/ml Wnt3a in presence or absence of 0.5 mg/ml Dkk1 or 5.4 mg/ml monomeric FL. The values of vehicle-treated cells at the start point on day 3 were set to 1. The data represent the mean of triplicates and standard deviation from one representative experiment. *p < 0.05 (vs Dkk1; significance was assessed by Student’s *t* test). **C–E** The incidence of spontaneously beating EBs derived from ES/scLRP6S/scFZD8L stimulated with vehicle, Wnt3a, or Wnt3a plus Dkk1 (**C**), with vehicle, BSA-FL, or BSA-FL plus Dkk1 (**D**), and with vehicle, BSA-FL, or BSA-FL plus FL (**E**) from day 6 to day 12 of differentiation. The data represent the mean and standard deviation from 3 to 10 independent experiments. *p < 0.05 (vs vehicle; significance was assessed by Turkey’s multiple comparison test)

To eliminate the possibility that the endogenous LRP6 and FZD8 influence the BSA-FL-induced signal activation in ES/scLRP6S/scFZD8L, we tested whether a Wnt3a antagonist, Dkk1, inhibits the BSA-FL-dependent induction of Wnt signaling. As Dkk1 antagonizes the Wnt3a signaling by competitive binding to the same extracellular region of LRP6 [5, 36], signal activation via scLRP6S, which has no extracellular domain of LRP6, should not be inhibited by Dkk1. As we expected, administration of high concentration of Dkk1 in ES/scLRP6S/scFZD8L markedly reduced endogenous Wnt3a signaling activity, while BSA-FL-induced signal transduction was not affected (Fig. 5A). We further tested the effects of Wnt inhibitors targeting intracellular Wnt signaling molecules. A tankyrase inhibitor, XAV939, which stabilizes the destruction complex, largely inhibited BSA-FL-induced TCF/β-catenin transactivation, whereas a porcupine inhibitor IWP-4, which blocks Wnt secretion, mildly decreased this transactivation (Fig. S8). These results suggest that BSA-FL directly transactivates canonical Wnt signaling. Moreover, FL monomers bind to an scFv region of the Wnt3a-diCAR and may competitively inhibit the signaling by preventing the complex formation of BSA-FL/scLRP6S/scFZD8L. Interestingly, BSA-FL-dependent signal activation in ES/scLRP6S/scFZD8L was prevented by addition of FL monomers in a dose-dependent manner (Fig. 5B). In addition, we analyzed the effects of FL monomers or Dkk1 on BSA-FL-mediated myocardial differentiation in EBs derived from ES/scLRP6S/scFZD8L. Wnt3a treatment promoted cardiomyocyte differentiation, which was abolished by Dkk1 (Fig. 5C). Similarly, BSA-FL treatment elevated the incidence of beating EBs (Fig. 5D). Although this increase was not influenced by Dkk1, monomeric FL significantly perturbed efficient induction of cardiomyocyte differentiation by BSA-FL (Fig. 5D and E). These data suggest that BSA-FL-dependent Wnt/ β-catenin signal activation in ES/scLRP6S/scFZD8L is mediated by the Wnt3a-diCARs and is involved in myocardial cell differentiation through an endogenous LRP6- and FZD8-independent pathway.

### TCF/β-catenin-dependent transcriptional activity induced by BSA-FL or Wnt3a displays similar characteristics during the differentiation process of ES/scLRP6S/scFZD8L

**Fig. 6:**
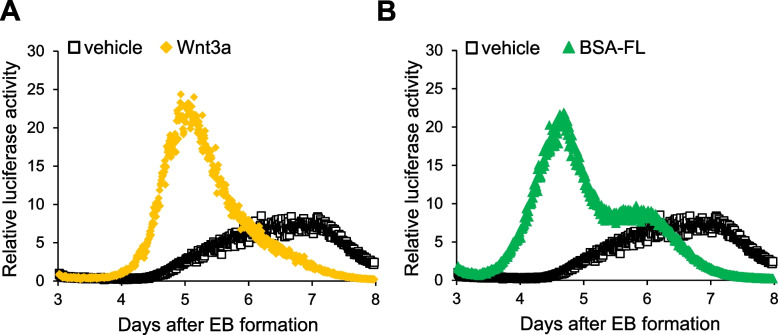
Real-time analysis of TCF/β-catenin-dependent transactivation in the course of differentiation from ES/scLRP6S/scFZD8L. **A, B** Representative traces from real-time luciferase analysis are shown. TCF/β-catenin-dependent transcriptional activities were measured every 10 min from day 3 to day 8 of differentiation in EBs derived from ES/scLRP6S/scFZD8L expressing the reporter gene. Cells were stimulated with vehicle, 0.1 μg/ml BSA-FL (**A**) or 50 ng/ml Wnt3a (**B**) from day 2 of differentiation for 24 h. The values of vehicle-treated cells at the start point on day 3 were set to 1

To further investigate the relationship between the efficiency of myocardial differentiation and the intensity of TCF/ β-catenin-dependent signaling, we performed a real-time luciferase reporter analysis. TCF/ β-catenin-dependent transcriptional activity during myocardial differentiation was measured in EBs derived from ES/scLRP6S/scFZD8L co-expressing luciferase reporter genes. The EBs were stimulated with BSA-FL, or Wnt3a from day 2 to day 3 of differentiation. The reporter activities were measured every 10 min from days 3 to 8. Interestingly, the maximum reporter intensity of the cells stimulated with BSA-FL was almost the same as that of Wnt3a-stimulated cells and both reporter activities reached their peak around day 5, after which they declined (Fig. 6A, B). In contrast, the reporter activities in the unstimulated cells were low in the early phase of differentiation and reached their peak at day 7. Considering the results previously reported [1, 2], this different timing of the maximum TCF/ β-catenin reporter activity may affect the efficiency of myocardial differentiation. Thus, stimulation by Wnt3a or BSA-FL in ES/scLRP6S/scFZD8L led to transient activation of canonical Wnt signaling in the early stage of differentiation, and subsequently promoted myocardial differentiation.

## Discussion

In the present study, we created functional artificial Wnt3a receptors, Wnt3a-diCAR, which can transduce the signals similar to canonical Wnt pathway. To begin the experiments, we constructed three types of chimeric LRP6 (scLRP6S, scLRP6M, and scLRP6L) and two types of chimeric FZD8 (scFZD8S and scFZD8L). Among the combinations of chimeric LRP6 and FZD8, scLRP6S/scFZD8S, and scLRP6S/scFZD8L appeared appropriate for establishing the stable ES cell lines, in which canonical Wnt signal is activated in response to the surrogate ligand, BSA-FL. Compared to the transient transfection (Fig. 1C), the stable expression of scLRP6S/scFZD8L exhibited TCF/ β-catenin-dependent transcription more strictly controlled by BSA-FL (Fig. 2A). In addition, the global changes of gene expression levels were similar between BSA-FL- and Wnt3a-treated cells in the early phase of differentiation (Fig. 2C, D). Consistent with previous studies [1, 2], canonical Wnt signal activation by either recombinant Wnt3a or BSA-FL in the early phase promoted myocardial cell differentiation (Figs. 3 and 4). Of note, the signal inhibition assay using Dkk1 and monomeric FL demonstrated that BSA-FL-responsive TCF/ β-catenin-dependent transactivation and enhanced myocardial differentiation were mediated by the signaling from the Wnt3a-diCAR but not from endogenous LRP6 and FZD8 (Fig. 5). This indicates that the Wnt3a-diCAR enables target-specific signal activation. Several studies reported that canonical Wnt signaling is inactivated and that non-canonical Wnt signal ligands, namely Wnt11 and Wnt5a, are involved in cardiac differentiation in the mid-to-late phase of embryonic development [1, 2, 37, 38]. This could be the reason explaining why continuous BSA-FL treatment (from days 2 to 6 of differentiation, as shown in Fig. 3B) did not increase the efficiency of beating cardiomyocyte production. In addition, compared to that with recombinant Wnt5a stimulation, BSA-FL did not seem to influence non-canonical Wnt signaling (Fig. S4). In this regard, BSA-FL stimulation might specifically activate canonical Wnt signaling. However, further studies are needed because cross-talk between canonical and non-canonical Wnt pathways has been reported, and they can be simultaneously activated.

We also examined the effects of single chimeric LRP6 or chimeric FZD8 on ligand-activated Wnt signal transduction. Each of the LRP6 chimeras activated the TCF/ β-catenin-dependent transcription without the ligands, while chimeric FZD8 alone showed almost no signal activation even with BSA-FL (Fig. S9). According to previous studies, LRP6 alone can transduce the signals without Wnt proteins and its signal activation is further enhanced by deletion of its extracellular domain [32, 39]. In addition, the extracellular domain of LRP6 was shown to repress the signal activity without ligands [40]. In this study, co-expression of scLRP6S and either scFZD8S or scFZD8L transduced the signal in a ligand-dependent manner. Consistently, a recent study demonstrated that surrogate Wnt agonists promotes the dimerization of LRP and FZD, which is required for Wnt signal transduction [41]. Taken together, our results also suggest that BSA-FL may promote hetero-dimerization of scLRP6 and scFZD8. Thus, it is interesting to further study spatiotemporal localization and/or conformational changes of scLRP6 and scFZD8 with or without BSA-FL. Therefore, the Wnt3a-diCARs are ultimately expected to contribute to the progression of the research on Wnt signal transduction as well as stem cell-based regenerative medicine. To date, human pluripotent stem cells have extensively been used in research on cancer biology, organ differentiation, and tissue regeneration, in which Wnt signaling is highly involved [9]. Hence, Wnt3a-diCAR-expressing human pluripotent stem cells are a promising tool to unveil canonical Wnt signaling functions in human cells.

Due to the hydrophobic property of Wnt3a, which is caused by its post-translational palmitoylation, the cost of production of recombinant Wnt proteins is very high, therefore hindering their further applications. Chemical Wnt agonists such as CHIR99021 are also expensive than BSA-FL. Recent advances of Wnt studies on adult stem cells have revealed pivotal roles of canonical Wnt signaling in tissue maintenance of hair follicles, mammary glands, hematopoietic systems, and gastrointestinal organs [9]. Recombinant Wnt3a protein indeed enables us to generate three-dimensional organoids derived from corresponding adult tissue stem cells. Therefore, the Wnt3a-diCAR is potentially applicable for activation of self-renewing adult stem cells, thereby promoting tissue regeneration when these cells are implanted with systemic administration of the surrogate ligand. In addition, BSA-FL is chemically stable and thus possesses its ligand activity even after 7 days of incubation in the culture medium (Fig. S10), without affecting other signaling pathways. Our study has the potential to boost in vivo application of Wnt3a-diCAR in tissue regeneration.

Accumulating evidence revealed that gene mutations in Wnt pathway components are frequently identified in cancer cells, and aberrant Wnt signal activation is considered as an oncogenic driver in several types of cancer [42, 43]. Thus, for regenerative medicine application, Wnt signaling needs to be strictly controlled within an appropriate range in target cells, even though its signal activity is modest. Recombinant Wnt3a protein and chemical canonical Wnt activators affect any cell types expressing canonical Wnt signaling molecules when they are systemically administered. In contrast, BSA-FL treatment can activate canonical Wnt signaling only in cells expressing Wnt3a-diCARs, which reduces the risk of adverse effects caused by systemic canonical Wnt signal activation. Moreover, monomeric FL would be helpful to antagonize the Wnt3a-diCAR-mediated signal activation, indicating the capability of fine-tuned on-off regulation. Taken together, our Wnt3a-diCAR would have advantages to achieve precise control of the canonical Wnt signal intensity.

Considering clinical applications, it would be necessary to establish suitable lines of human pluripotent stem cells that sufficiently express Wnt3a-diCARs and highly respond to surrogate ligands, whereas we confirmed that BSA-FL-induced canonical Wnt signaling can be activated in several lines of Wnt3a-diCAR-expressing mouse ES cells (Fig. S11). Further testing to determine whether these cell lines contain tumorigenic mutations should also be carried out for safety validation. In this study, Wnt3a-diCARs were introduced into ES cells via lentiviral vectors, which is a highly efficient way to test cellular functions of BSA-FL-diCAR-dependent signaling. Although lentiviral transgene integration enables long-term gene expression, there are several concerns for clinical use, including genomic instability, tumorigenesis, and interclonal variation among cell lines. Thus, a transient expression system is safer and helpful, and gene delivery systems using non-genome-integrating methods (e.g., adeno-associated virus, sendaivirus, transfection reagents) would be desirable for a diCAR-dependent regeneration therapy. These issues will be solved in future studies, which will lead to research advancements in regenerative medicine in humans.

## Conclusion

In the present study, we, for the first time, developed GPCR-based CARs. The Wnt3a-diCAR successfully activates endogenous Wnt3a signaling in response to BSA-FL, a surrogate ligand, which is chemically stable and inexpensive to produce. As one example of the utilities, we show that the Wnt3a-diCAR would be available to generate a sufficient number of cardiomyocytes differentiated from pluripotent stem cells with low costs. In addition, the Wnt3a-diCAR might be a valuable tool to analyze the Wnt signaling pathway. Further studies contributing to better control of the canonical Wnt signaling would facilitate the application of this technology in the clinic for regenerative medicine.

## Supplementary Information

**Additional file 1: Fig. S1.** TCF/β-catenin-dependent transcriptional activities of mouse ES cells stably expressing scLRP6S and scFZD8S. TCF/β-catenin-dependent luciferase activities in ES/mock and ES/scLRP6S/scFZD8S following stimulation with BSA-FL are shown. Luciferase luminescence intensities were normalized to Renilla luciferase luminescence intensities derived from co-transfected pSV40-Rluc. The values of mock-transduced cells treated with vehicle were set to 1. The data represent the mean of triplicates and standard deviation from one representative experiment. **Fig. S2.** TCF/β-catenin-dependent transcriptional activities in mouse ES cells stably expressing scLRP6S and scFZD8L. TCF/β-catenin-dependent luciferase activities in ES/mock and ES/scLRP6S/scFZD8L cells following stimulation with CHIR99021 (a WNT activator) or BSA-FL at indicated concentrations are shown. Luciferase luminescence intensities were normalized to *Renilla* luciferase luminescence intensities derived from co-transfected pSV40-Rluc. The values of mock-transduced cells treated with vehicle were set to 1. The data represent the mean and standard deviation. **Fig. S3.** TCF/β-catenin-dependent transcriptional activities of mouse embryonic fibroblasts (MEFs) expressing scLRP6S and scFZD8L. TCF/β-catenin-dependent luciferase activities in MEFs expressing mock or scLRP6S/scFZD8L following stimulation with BSA-FL at the indicated concentrations are shown. Luciferase luminescence intensities were normalized to *Renilla* luciferase luminescence intensities derived from co-transfected pSV40-Rluc. The values of mock-transduced cells treated with vehicle were set to 1. The data represent the mean and standard deviation from triplicate measurements. **Fig. S4.** Western blotting for non-canonical Wnt activation in scLRP6S and scFZD8L-expressing cells. Protein expression levels of Ror2 and β-actin in mock- or scLRP6S/scFZD8L-expressing ES cells (A) or NIH3T3 cells (B) treated with Wnt5a, Wnt3a or BSA-FL are shown. **Fig. S5.** BSA-FL diffuses into EBs. Representative fluorescence microscopy images in EB sections from ES/mock and ES/scLRP6S/scFZD8L cells treated with vehicle or BSA-FL. Nuclei are counterstained with DAPI showing BSA-FL deposition possibly in the plasma membrane. The right panels are enlarged images of the boxed area of the BSA-FL-treated EBs. Scale bars = 50 μm. **Fig. S6.** Incidence of spontaneously beating EBs derived from three independent ES cell lines expressing scLRP6S and scFZD8L. The incidence of spontaneously beating EBs stimulated by 0.1 μg/ml BSA-FL, 50 ng/ml Wnt3a, or vehicle for 24 h from day 2 of differentiation is shown. Time course of the incidence of spontaneously beating EBs differentiated from ES/scLRP6S/scFZD8L stimulated by 0.1 μg/ml BSA-FL, 50 ng/ml Wnt3a, or vehicle for 24 h from day 2 after EB formation. **Fig. S7.** BSA-FL enhances cardiac differentiation of EBs expressing scLRP6S and scFZD8L. Representative flow cytometry plots of cardiac troponin T (cTnT, indicated by FITC) vs. phycoerythrin (PE) for differentiated ES/mock and ES/scLRP6S/scFZD8L treated with vehicle, Wnt3a or BSA-FL. **Fig. S8.** TCF/β-catenin-dependent transcriptional activities in mouse ES cells stably expressing scLRP6S and scFZD8L. TCF/β-catenin-dependent luciferase activities in ES/mock and ES/scLRP6S/scFZD8L cells following BSA-FL treatment with or without IWP-4 or XAV939, WNT inhibitors, are shown. Luciferase luminescence intensities were normalized to *Renilla* luciferase luminescence intensities derived from co-transfected pSV40-Rluc. The values of mock-transduced cells treated with vehicle were set to 1. The data represent the mean and standard deviation. **Fig. S9.** TCF/β-catenin-dependent transcriptional activities in mouse ES cells expressing single chimeric receptors. TCF/β-catenin-dependent transcriptional activities measured by luciferase reporter assay in mouse ES cells transiently transfected with plasmids encoding various chimeric LRP6 or FZD8. The values in mock-transfected cells treated with vehicle were set to 1. The data represent the mean of duplicates and standard deviation from one representative experiment. **Fig. S10.** Activity of BSA-FL and Wnt3a after incubation at 37 °C. TCF-dependent transcriptional activities were measured by luciferase reporter assay in mouse ES cells transiently transfected with scLRP6S and scFZD8L. The cells were stimulated with 0.1, 1, or 10 μg/ml BSA-FL or 50 ng/ml Wnt3a that were incubated at 37 °C for 0, 1, 2, 4, or 7 days. Luciferase activities of samples stimulated by each ligand with no incubation were set to 100. **Fig. S11.** TCF/β-catenin-dependent transcriptional activities in three independent mouse ES cell lines stably expressing scLRP6S and scFZD8L. TCF/β-catenin-dependent luciferase activities in ES/scLRP6S/scFZD8L following indicated BSA-FL treatment are shown. Luciferase luminescence intensities were normalized to *Renilla* luciferase luminescence intensities derived from co-transfected pSV40-Rluc. The data represent the mean and standard deviation. **Table S1.** List of primer sequences used for plasmid construction. **Table S2.** List of primer sequences used for real-time qPCR.

**Additional file 2: Movie S1.** Calcium oscillations in differentiated ES/scLRP6S/scFZD8L. Calcium oscillations, monitored by using Fluo-8 AM calcium indicator, were observed on day 17 of differentiation in ES/scLRP6S/scFZD8L stimulated with 1 μg/ml BSA-FL from day 2 to day 3**.**

## Data Availability

The datasets used and/or analyzed during the current study are available from the corresponding author on reasonable request.

## Abbreviations

aiCAR: Apoptosis-inducing chimeric antigen receptor
BSA: Bovine serum albumin
CRD: Cysteine-rich domain
cTnT: Cardiac troponin T
diCAR: Differentiation-inducing chimeric receptor
ES: Embryonic stem
FL: Fluorescein
FZD8: Frizzled 8
GPCR: G protein-coupled receptor
LRP6: Lipoprotein receptor-related protein 6
MEF: Mouse embryonic fibroblast
miCAR: Migration-inducing chimeric antigen receptor
piCAR: Proliferation-inducing chimeric antigen receptor
scFv: Single-chain Fv
TCF: T cell factor
TM: Transmembrane
