# Supplementary material for: Canonical Wnt signaling activation by chimeric antigen receptors for efficient cardiac differentiation from mouse embryonic stem cells
Source: Inflamm Regen. 2023 Feb 10;43:11. doi: 10.1186/s41232-023-00258-6 (PMC9912504; doi:10.1186/s41232-023-00258-6)
Supplement: Supplementary file 1 — Additional file 1: Fig. S1. TCF/β-catenin-dependent transcriptional activities of mouse ES cells stably expressing scLRP6S and scFZD8S. TCF/β-catenin-dependent luciferase activities in ES/mock and ES/scLRP6S/scFZD8S following stimulation with BSA-FL are shown. Luciferase luminescence intensities were normalized to Renilla luciferase luminescence intensities derived from co-transfected pSV40-Rluc. The values of mock-transduced cells treated with vehicle were set to 1. The data represent the mean of triplicates and standard deviation from one representative experiment. Fig. S2. TCF/β-catenin-dependent transcriptional activities in mouse ES cells stably expressing scLRP6S and scFZD8L. TCF/β-catenin-dependent luciferase activities in ES/mock and ES/scLRP6S/scFZD8L cells following stimulation with CHIR99021 (a WNT activator) or BSA-FL at indicated concentrations are shown. Luciferase luminescence intensities were normalized to Renilla luciferase luminescence intensities derived from co-transfected pSV40-Rluc. The values of mock-transduced cells treated with vehicle were set to 1. The data represent the mean and standard deviation. Fig. S3. TCF/β-catenin-dependent transcriptional activities of mouse embryonic fibroblasts (MEFs) expressing scLRP6S and scFZD8L. TCF/β-catenin-dependent luciferase activities in MEFs expressing mock or scLRP6S/scFZD8L following stimulation with BSA-FL at the indicated concentrations are shown. Luciferase luminescence intensities were normalized to Renilla luciferase luminescence intensities derived from co-transfected pSV40-Rluc. The values of mock-transduced cells treated with vehicle were set to 1. The data represent the mean and standard deviation from triplicate measurements. Fig. S4. Western blotting for non-canonical Wnt activation in scLRP6S and scFZD8L-expressing cells. Protein expression levels of Ror2 and β-actin in mock- or scLRP6S/scFZD8L-expressing ES cells (A) or NIH3T3 cells (B) treated with Wnt5a, Wnt3a or BSA-FL are shown. Fig [file 41232_2023_258_MOESM1_ESM.pdf]

Supplemental Information

Canonical Wnt signaling activation by chimeric antigen receptors for efficient cardiac differentiation from mouse embryonic stem cells

Takahiro Sogo, Tasuku Tsukamoto, Shu Nakao, Yukihiro Harada, Dai Ihara, Tomoe Ueyama, Tomoaki Ishida, Masato Nakahara, Koji Hasegawa, Yuka Akagi, Yasuyuki S Kida, Osamu Nakagawa, Teruyuki Nagamine, Masahiro Kawahara, Teruhisa Kawamura

Fig. S1

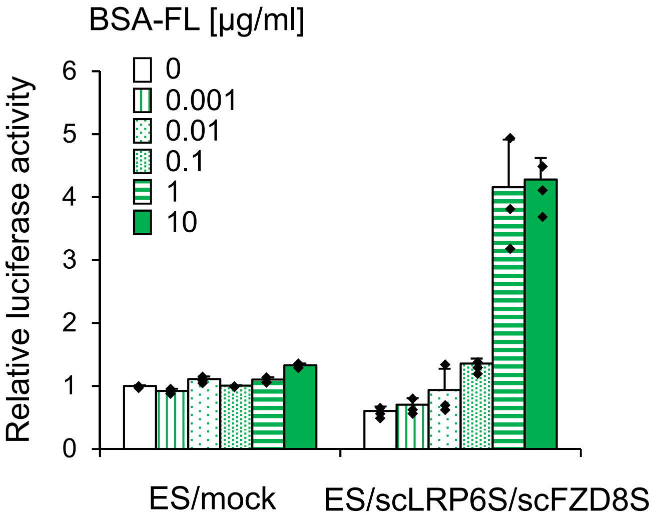

Fig. S1. TCF/β-catenin-dependent transcriptional activities of mouse ES cells stably expressing scLRP6S and scFz8S.

TCF/β-catenin-dependent luciferase activities in ES/mock and ES/scLRP6S/scFZD8S following stimulation with BSA-FL are shown. Luciferase luminescence intensities were normalized to Renilla luciferase luminescence intensities derived from co-transfected pSV40-Rluc. The values of mock-transduced cells treated with vehicle were set to 1. The data represent the mean and standard deviation from 3 independent experiments.

Fig. S2

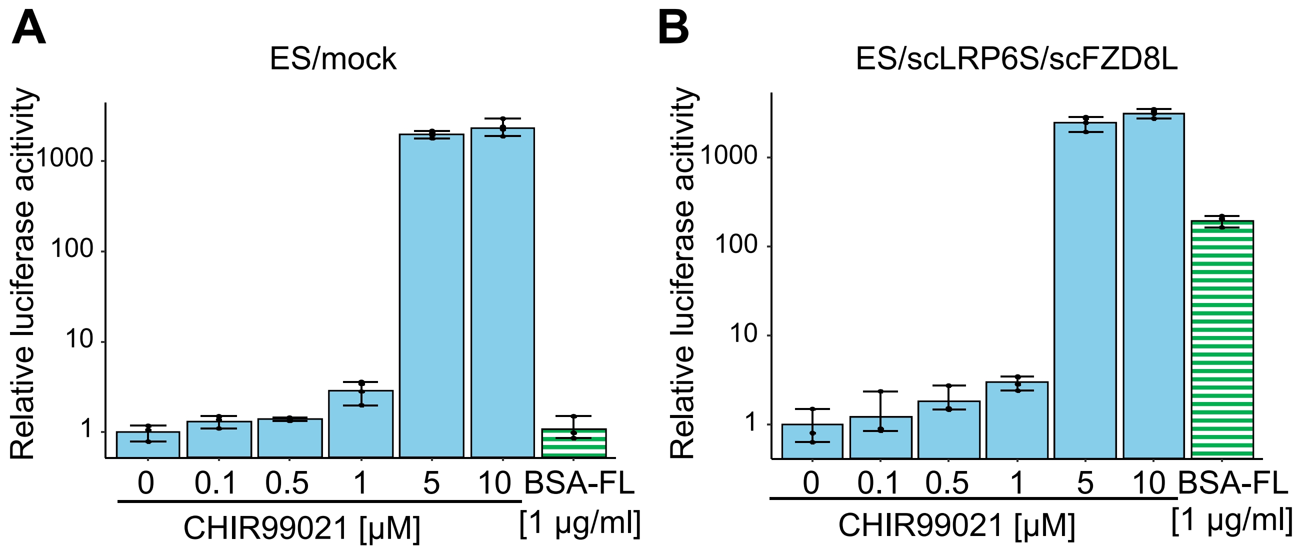

Fig. S2. TCF/β-catenin-dependent transcriptional activities in mouse ES cells stably expressing scLRP6S and scFZD8L.

TCF/β-catenin-dependent luciferase activities in ES/mock and ES/scLRP6S/scFZD8L following stimulation with CHIR99021 (a WNT activator) or BSA-FL at indicated concentrations are shown. Luciferase luminescence intensities were normalized to Renilla luciferase luminescence intensities derived from co-transfected pSV40-Rluc. The values of mock-transduced cells treated with vehicle were set to 1. The data represent the mean and standard deviation.

Fig. S3

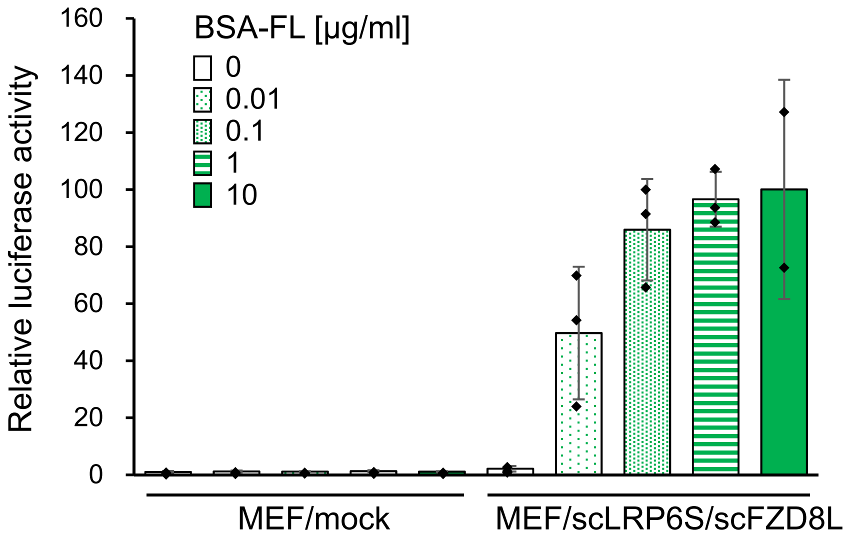

Fig. S3. TCF/β-catenin-dependent transcriptional activities of mouse embryonic fibroblasts (MEFs) expressing scLRP6S and scFZD8L.

TCF/β-catenin-dependent luciferase activities in MEFs expressing mock or scLRP6S/scFZD8L following stimulation with BSA-FL at the indicated concentrations are shown. Luciferase luminescence intensities were normalized to Renilla luciferase luminescence intensities derived from co-transfected pSV40-Rluc. The values of mock-transduced cells treated with vehicle were set to 1. The data represent the mean and standard deviation from triplicates.

Fig. S4

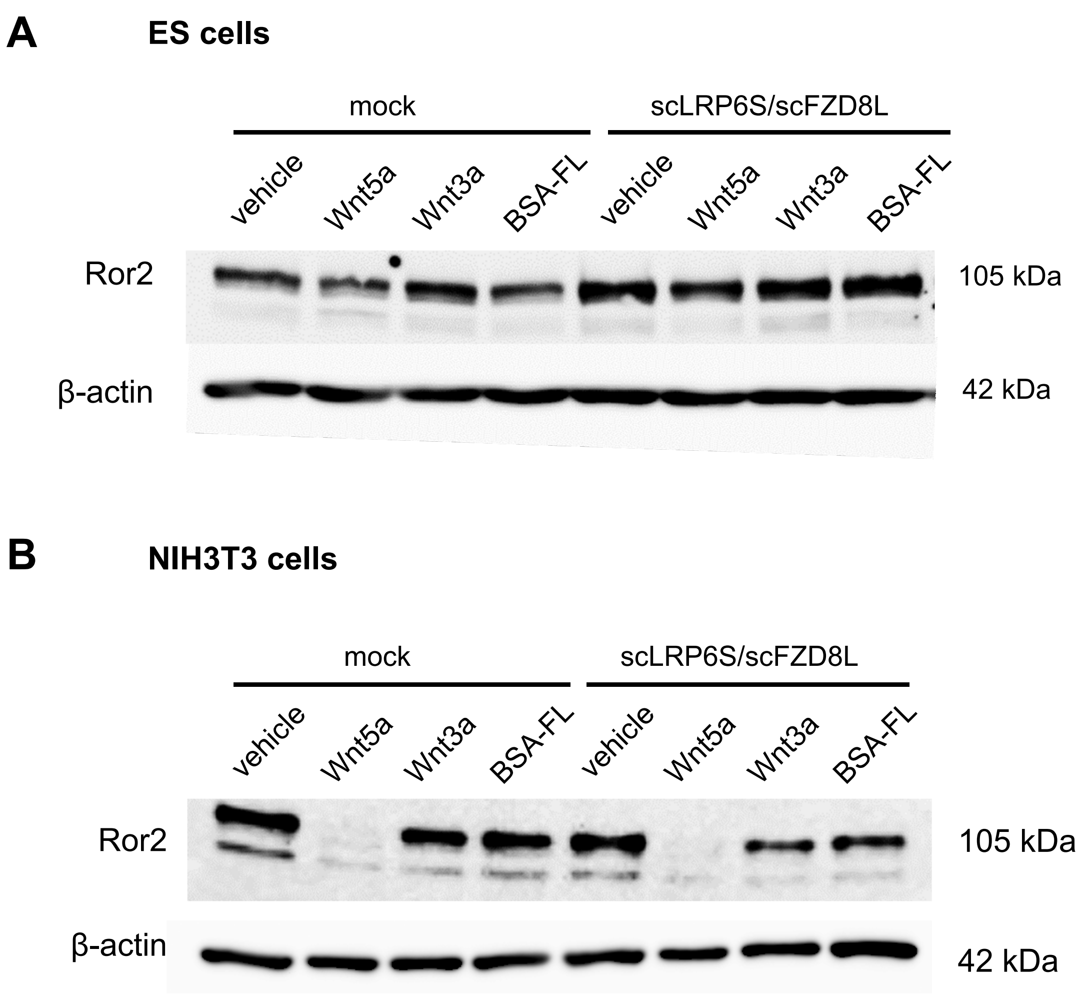

Fig. S4. Western blotting for non-canonical Wnt activation in scLRP6S and scFZD8L-expressing cells.

Protein expression levels of Ror2 and β-actin in mock- or scLRP6S/scFZD8L -expressing ES cells (A) or NIH3T3 cells (B) treated with Wnt5a, Wnt3a or BSA-FL are shown.

Fig. S5

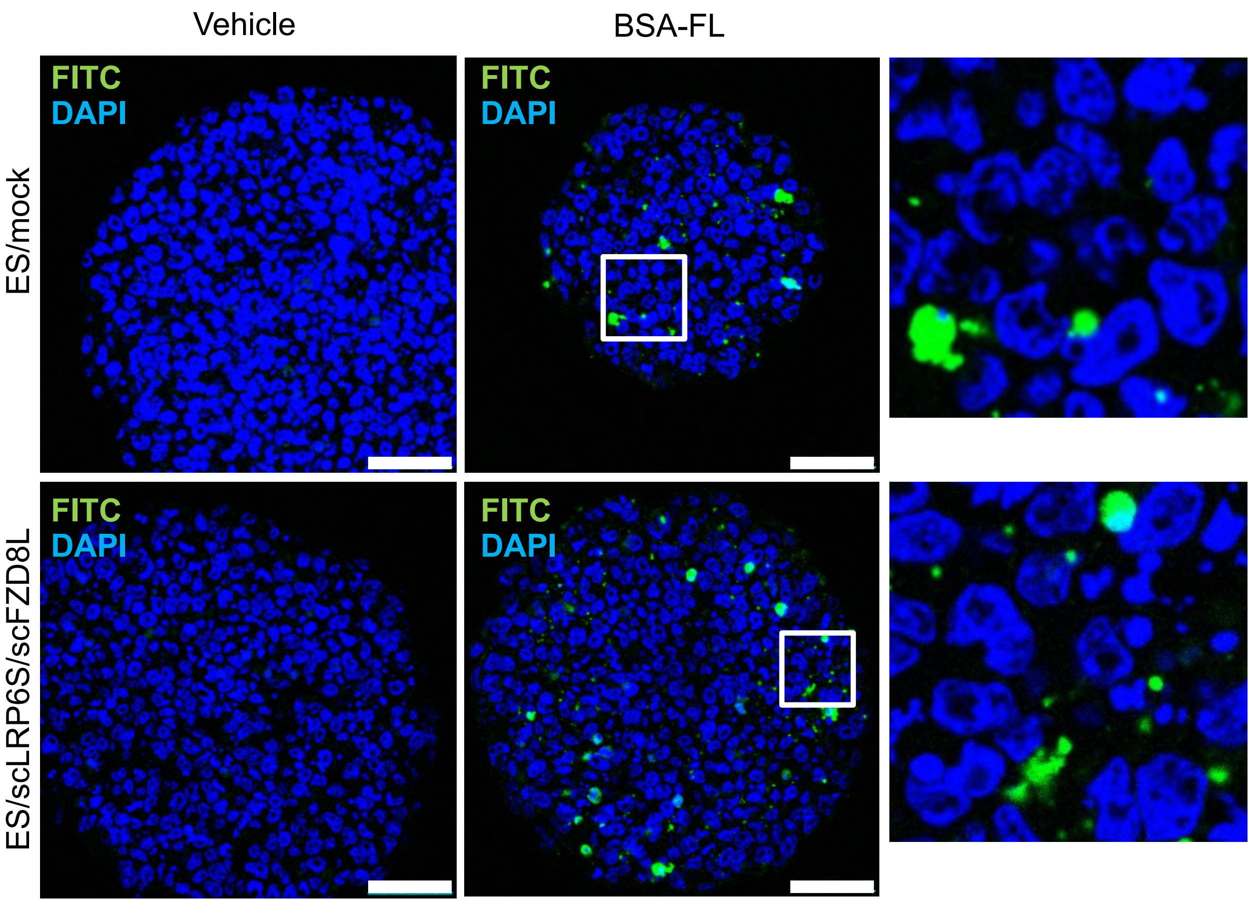

Fig. S5. BSA-FL is diffused into EBs.

Representative fluorescence microscopy images in EB sections from ES/mock and ES/scLRP6S/scFZD8L treated with vehicle or BSA-FL. Nuclei are counterstained with DAPI showing BSA-FL deposition possibly in the plasma membrane. The right panels are enlarged images of the boxed area of the BSA-FL-treated EBs. Scale bars = 50 μm.

Fig. S6

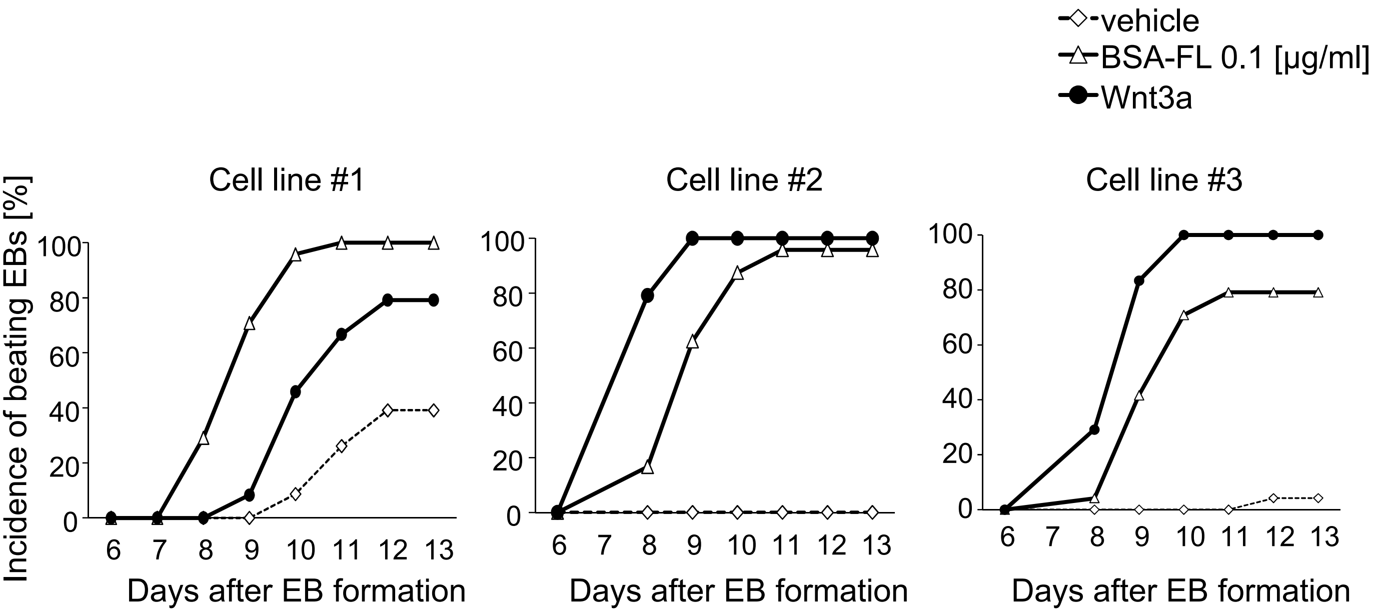

Fig. S6. The incidence of spontaneously beating EBs derived from three independent ES cell lines expressing scLRP6S and scFZD8L.

The incidence of spontaneously beating EBs stimulated by 0.1 μg/ml BSA-FL, 50 ng/ml Wnt3a, or vehicle for 24 hours from day 2 of differentiation is shown.

Fig. S7

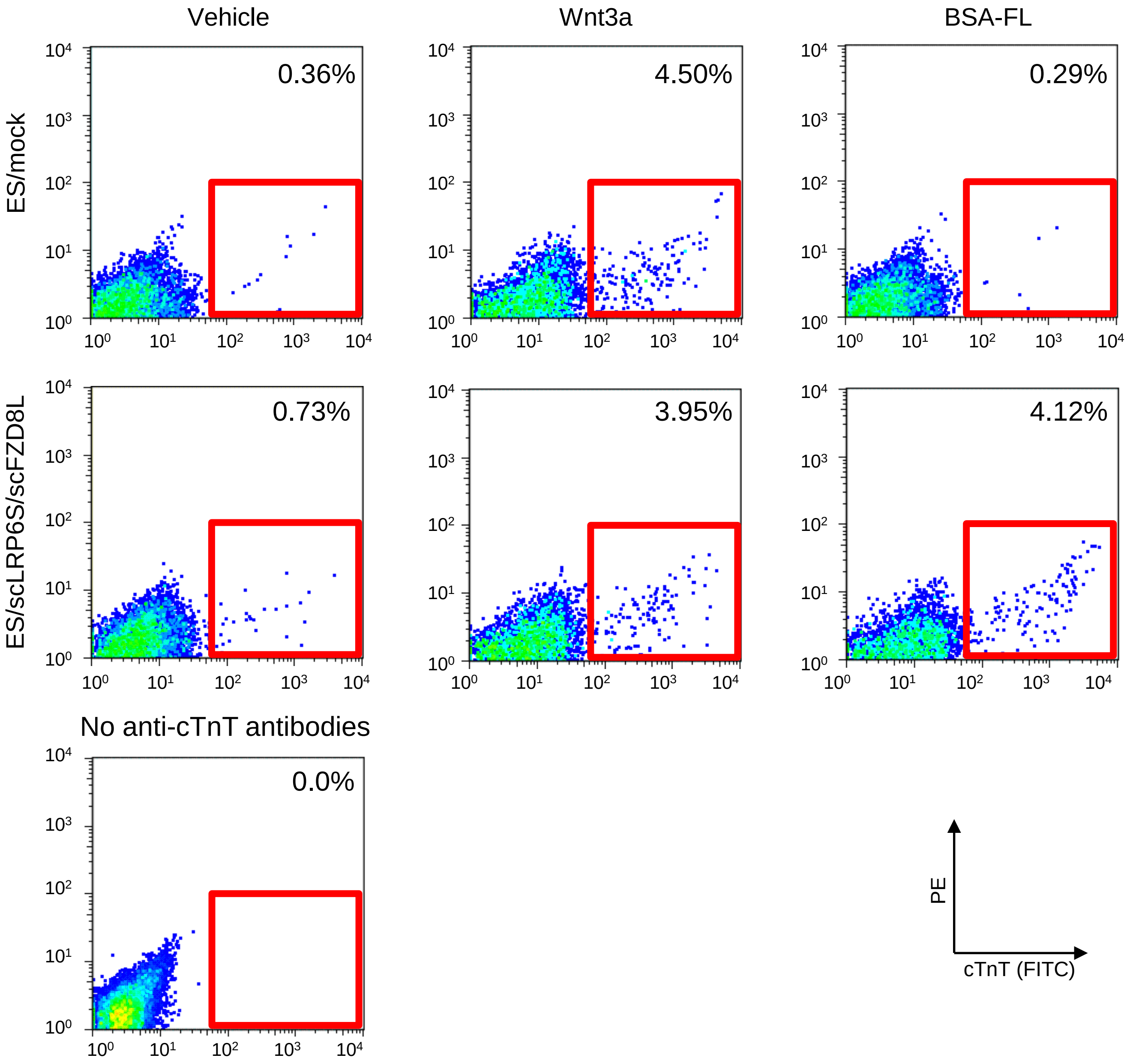

Fig. S7. BSA-FL enhances cardiac differentiation of EBs expressing scLRP6S and scFZD8L.

Representative flow cytometry plots of cardiac troponin T (cTnT, indicated by FITC) vs. phycoerythrin (PE) for differentiated ES/mock and ES/scLRP6S/scFZD8L treated with vehicle, Wnt3a or BSA-FL.

Fig. S8

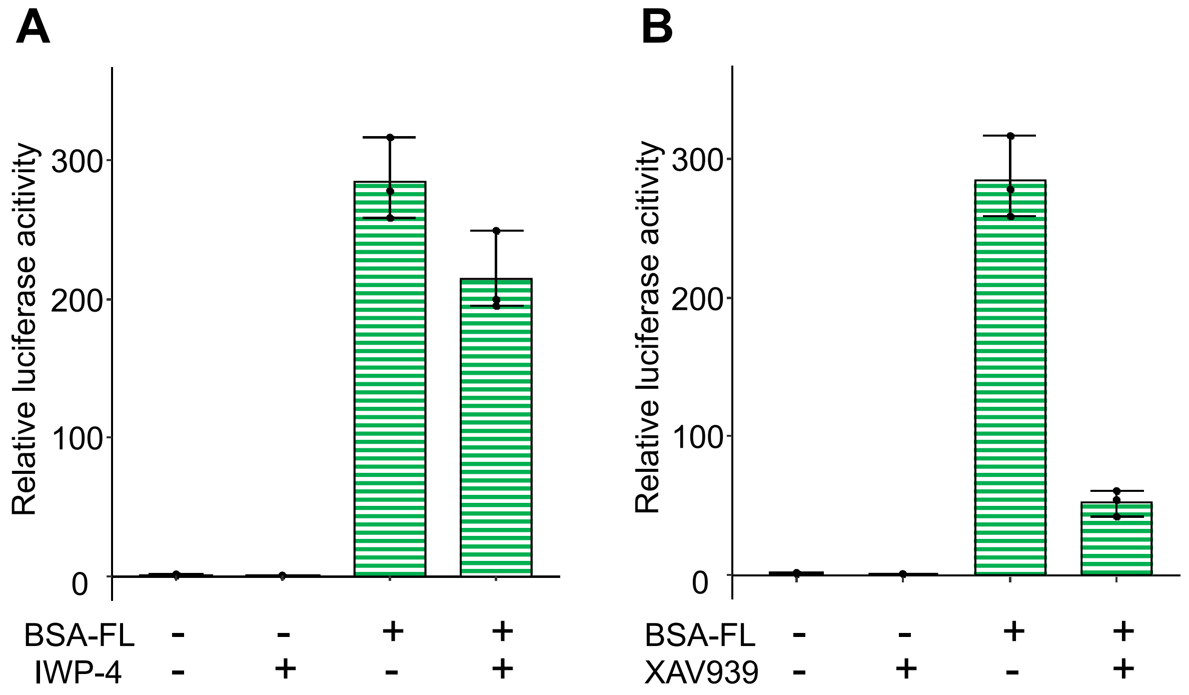

Fig. S8. TCF/β-catenin-dependent transcriptional activities in mouse ES cells stably expressing scLRP6S and scFZD8L.

TCF/β-catenin-dependent luciferase activities in ES/mock and ES/scLRP6S/scFZD8L following BSA-FL treatment with or without IWP-4 or XAV939, WNT inhibitors, are shown. Luciferase luminescence intensities were normalized to Renilla luciferase luminescence intensities derived from co-transfected pSV40-Rluc. The values of mock-transduced cells treated with vehicle were set to 1. The data represent the mean and standard deviation.

Fig. S9

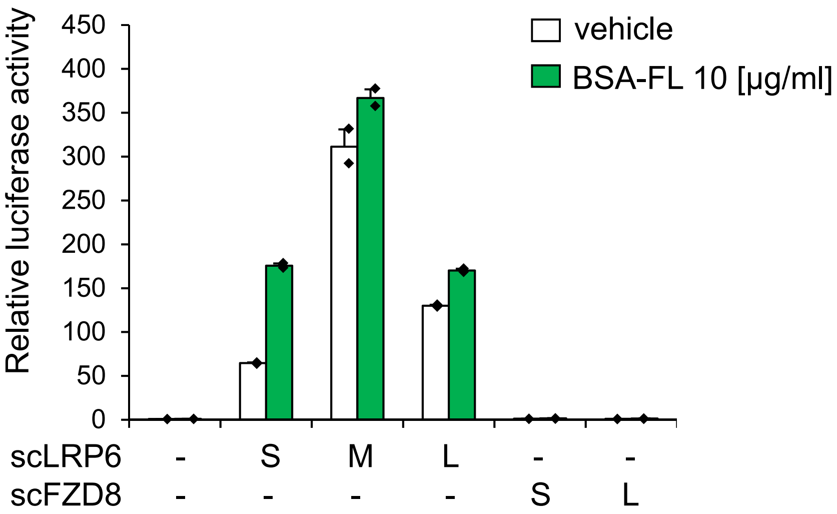

Fig. S9. TCF/β-catenin-dependent transcriptional activities in mouse ES cells expressing single chimeric receptors.

TCF/β-catenin-dependent transcriptional activities measured by luciferase reporter assay in mouse ES cells transiently transfected with plasmids encoding various chimeric LRP6 or FZD8. The values in mock-transfected cells treated with vehicle were set to 1. The data represent the mean of duplicates and standard deviation from one representative experiment.

Fig. S10

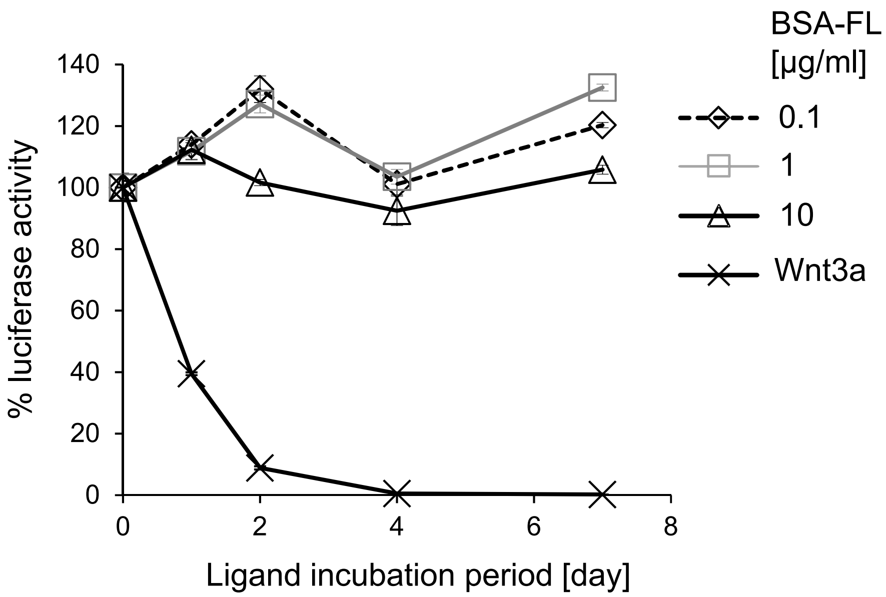

Fig. S10. Activity of BSA-FL and Wnt3a after incubation at 37 ° C .

TCF-dependent transcriptional activities were measured by luciferase reporter assay in mouse ES cells transiently transfected with scLRP6S and scFZD8L. The cells were stimulated with 0.1, 1, or 10 μg/ml BSA-FL or 50 ng/ml Wnt3a that were incubated at 37 ° C for 0, 1, 2, 4, or 7 days. Luciferase activities of samples stimulated by each ligand with no incubation were set to 100.

Fig. S11

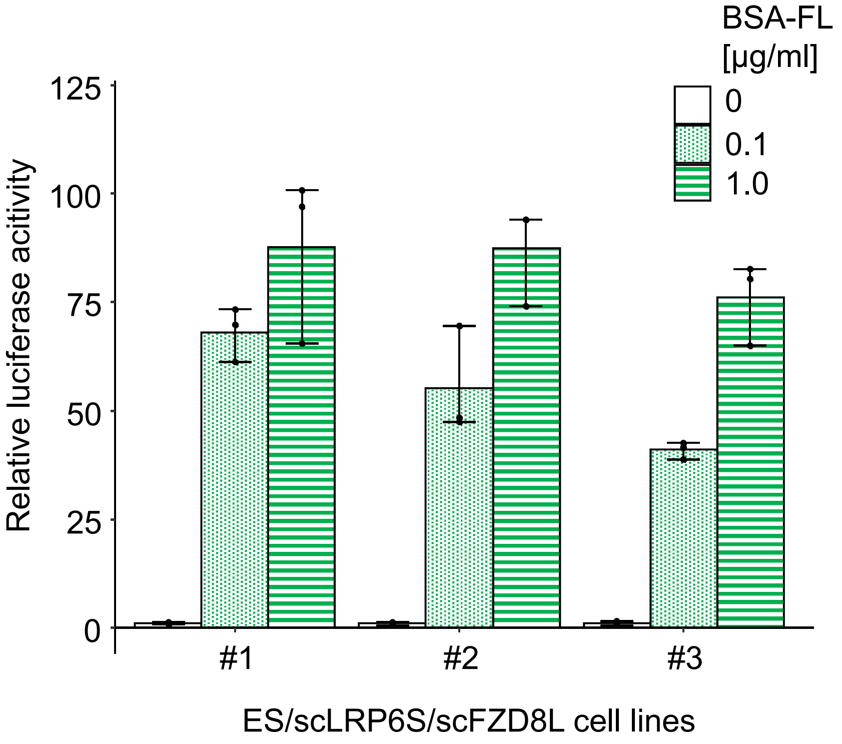

Fig. S11. TCF/β-catenin-dependent transcriptional activities in three independent mouse ES cell lines stably expressing scLRP6S and scFZD8L.

TCF/β-catenin-dependent luciferase activities in ES/scLRP6S/scFZD8L following indicated BSA-FL treatment are shown. Luciferase luminescence intensities were normalized to Renilla luciferase luminescence intensities derived from co-transfected pSV40-Rluc. The data represent the mean and standard deviation.

Table S1. List of primer sequences used for plasmid construction.

Primer name	Forward primer (5' to 3')	Reverse primer (5' to 3')
scFv	GCCTTCGAAACCATGGGATGGAGCTGTATC	GCCGATATCCGGAACCTGCAGTCAAGAG
LRP6L	CGGTCCCCGAGGCTTTCCTTCTG	TCAGGAGGAGTCCGTGCAGGG
LRP6M	CGACGTGTTCTCCTCAGCAGTTTACC	TCAGGAGGAGTCCGTGCAGGG
LRP6S	GCCACCGGTACCAACACAGTTGGTTCTGTTATTGG	GCCACCGGTCAGGAGGAGTCCGTGCAGGG
FZD8L	CGAACCGCACCGACCTCACCACG	TCAGACCTGGGACAATGGCATTTGC
FZD8S	GCCACCGGTTTCACCGTCTTCTGGATCGG	GCCACCGGTCAGACCTGGGACAATGGCATTTGC

Table S2. List of primer sequences used for quantitative RT-PCR.

Gene	Forward primer (5' to 3')	Reverse primer (5' to 3')
Gapdh	AGGTCGGTGTGAACGGATTTG	TGTAGACCATGTAGTTGAGGTCA
Axin2	TGACTCTCCTTCCAGATCCCA	TGCCCACACTAGGCTGACA
Epha1	CCGAGGAAGTCACTCTAATGGA	GGGGTGTCCCGTTTAGCAT
Fgf8	GAGACCGATACTTTTGGAAGCA	TCTCTGTGAATACGCAGTCCT
Fst	GAGCAAGGAAGAGTGTTGCAG	CTCACACGTTTCTTTACAAGGGA
Wnt8a	TCCAGACTCTTCGTGGACAGT	CAGGTCCTTTTCGTGGAGGC
Gata4	CGCCGGTTTTCTGGGAA	CACCGGCTAAAGAAGCCTA
Isl1	GGAAGAAACCAGCCTCAGTG	AGGATGGGAGGAGAGGCAA
Mesp1	CTACCCTAGACCCCGAGTC	CGGCGTCCAGGTTTCTAG
Gata6	ACCAGCAGCGACTAGCA	GGCGCTACTCCAACCTGA
Flk1	GGCGGTGGTGACAGTATCTT	CTCGGTGATGTACACGATGC
Sox17	GCTCCTGCTTTTGGTGTAGC	GTCCTTGGGCAGTCATTCAT
Myh7	CTACAGGCCTGGGCTTACCT	TCTCCTTCTCAGACTTCCGC
Myh6	GAGATTTCTCCAACCCAG	TCTGACTTTCGGAGGTACT
Actc1	CCAGCCCAGCTGAATCC	CCATTGTCACACACCAAAGC
Cdh5	AGACACCCCCAACATGCTAC	GCAAACTCTCCTTGGAGCAC
Cata3	CTCGGCCATTCGTACATGGAA	GGATACCTCTGCACCGTAGC
Acta2	CTGACAGAGGCACCACTGAA	AGAGGCATAGAGGGACAGCA
Gata1	AGCATCAGCACTGGCCTACT	AGGCCCAGCTAGCATAAGGT
Osx	ATGGCGTCCTCTCTGCTTG	TGAAAGGTCAGCGTATGGCTT
Runx2	CGGCCCTCCCTGAACTCT	TGCCTGCCTGGGATCTGTA
Afp	TCCAGAAGGAAGAGTGGACAA	GCAGACTAGGAGAAGAGAAATAGTTGA
Pdx1	CCCCAGTTTACAAGCTCGCT	CTCGGTTCCATTCGGGAAAGG
Pax6	AAGGAGGGGGAGAGAACACC	TCTGAGCTTCATCCGAGTCTT
Nestin	CCCTGAAGTCGAGGAGCTG	CTGCTGCACCTCTAAGCGA
Neurod1	ATGACCAAATCATACAGCGAGAG	TCTGCCTCGTGTTCCTCGT
